# S-Nitrosoglutathione protects acute kidney injury in septic rats by inhibiting the activation of NLRP3 inflammasome

**DOI:** 10.22038/IJBMS.2023.69651.15169

**Published:** 2023

**Authors:** Heng Fan, Min Sun, Jian-wei Le, Jian-hua Zhu

**Affiliations:** 1 Department of Intensive Care Unit, The First Affiliated Hospital of Ningbo University, Ningbo, Zhejiang Province, P.R China

**Keywords:** Acute kidney injury, Nucleotide oligomerization - domain-like receptor protein3, Rat, Sepsis, S-Nitrosoglutathione

## Abstract

**Objective(s):**

We aimed to study the effect of S-nitroso glutathione (SNG) on acute kidney injury (AKI) in septic rats by regulating nucleotide oligomerization domain-like receptor protein 3 (NLRP3).

**Materials and Methods:**

Sprague Dawley rats were used to construct the AKI model, and biochemical methods were used to detect the levels of inflammatory factors and anti-oxidant enzymes in renal tissue. We observed the ultrastructural changes of renal tissue by transmission electron microscopy and detected the protein and mRNA levels of NLRP3, apoptosis-associated speck-like protein containing a caspase recruitment domain foci (ASC) and caspase-1 by western-blotting and RT-qPCR.

**Results:**

Cecal ligation and puncture induced renal tubular epithelial tissue damage in septic rats, resulting in decreased renal function, increased levels of inflammation and decreased levels of anti-oxidant enzymes in renal tissue, and aggravated mitochondrial damage, significantly decreased mitochondrial density and enzyme complex I/II/III/IV levels (all *P*<0.001), and increased the protein and mRNA expression of NLRP3, ASC, and caspase-1 (all *P*<0.001). However, after pretreatment with SNG, the pathological damage of renal tubular epithelial tissue was reduced, the renal function was improved, the level of inflammation in renal tissue decreased and the level of anti-oxidant enzymes increased, the density of mitochondria and the level of enzyme complex I/II/III/IV were significantly increased (all *P*<0.001), meanwhile the protein and mRNA levels of NLRP3, ASC, and caspase-1 were all decreased significantly (all *P*<0.05).

**Conclusion:**

SNG protects AKI in septic rats by inhibiting NLRP3 inflammasome activation.

## Introduction

Sepsis is caused by the imbalance of inflammatory reactions in the body, resulting in fatal organ damage and dysfunction, and the mortality rate is as high as 60% (1-3). Septic acute kidney injury (AKI) accounts for about 30–60% of all causes of AKI, and it has the characteristics of high morbidity and mortality (4). It increases the risk of chronic and/or end-stage kidney disease **(**1**)**. Currently, it mainly relies on renal replacement therapy and lacks effective early treatment drugs (5).

Nephritis plays a dominant role in septic AKI, and activation of the nucleotide oligomerization domain-like receptor protein 3 (NLRP3) inflammasome is critical for the development of nephritis (6). Previous studies found that S-nitroso glutathione (SNG) has anti-inflammatory and anti-oxidant effects, which decrease the mortality of septic rats (7, 8). Meanwhile, studies showed that SNG also has important anti-inflammatory and organ protection effects (9, 10). In the present study, we will establish a rat model of septic AKI by cecal ligation and puncture (CLP), and investigate the effect of SNG on septic AKI and its regulatory mechanisms on the activation of NLRP3 inflammasome.

## Materials and Methods

***Animals and grouping***

Forty clean-grade Sprague Dawley (SD) rats, 250±20 g body weight, 8–10 weeks old, purchased and bred in Ningbo University, housed at relative humidity 45%–55%, room temperature 22–25 ℃, 12 hr day and night alternation, with free access to water and food. We randomly divided them into 4 groups: Control (n=10), Sham (n=10), CLP (n=10), and CLP+SNG (n=10) group. We used the CLP method to prepare the rat model (9). Specific steps: The rats were anesthetized by intraperitoneal injection of 2% pentobarbital sodium (0.1 ml/20 g). The skin was prepared and disinfected, the abdominal cavity was opened, the cecum was found, the root of the cecum was ligated, three holes were punched in the root of the cecum, a small amount of feces was squeezed out, and the incision was sutured. The control group did not receive any treatment, while the rats in the Sham group were operated on and off the abdominal cavity. Rats in the CLP+SNG group were infused with SNG at a rate of 50 µg/kg/h through the tail vein 1 hr before CLP. All our operations complied with animal ethics and were approved by the Animal Ethics Committee (ID: AEWC-2017-33).

***Determination of renal function***

The rats were killed by spinal dislocation 48 hr after CLP and the whole blood was collected by cardiac puncture. We used an automatic biochemical analyzer to detect the renal function indicators of rats, such as plasma kidney injury molecule-1 (pKIM-1), blood urea nitrogen (BUN), Serum creatinine (SCr), and plasma neutrophil gelatinase-related lipid transporter (pNGAL). 

***Renal histopathology***

The renal tissue was taken and immersed in 4% paraformaldehyde for 24 hr. After dehydration, transparency, and paraffin embedding, it was cut into 4 µm thick sections. Hematoxylin-eosin (H&E) staining was used to observe the pathological changes under a light microscope (400×). According to our previous experience, the semi-quantitative score of renal tubular injury was: 0 for no injury; 1 point for damage degree <25%; 2 points if 25%≤ damage degree <50%; 50%≤ damage degree <75%: 3 points; ≥ 75% is 4 points (8).

***Determination of inflammatory factors***

We used an enzyme-linked immunosorbent assay (ELISA) kit (Jinqiao Biotechnology, Beijing, China) to determine the levels of interleukin (IL)-8 and IL-6, and tumor necrosis factor-α (TNF-α). We added 50 µl of standard solution to the standard hole according to the instructions to establish the standard curve, added 40 µl sample diluent and 10 µl sample to the sample hole for subsequent operation, measured the absorbance value of each hole, and drew the standard curve.

***Determination of anti-oxidant enzyme***

According to the instructions of the kit (Enhua Biotechnology, Suzhou, China), we used biochemical methods to measure the levels of glutathione peroxidase (GSH-Px), catalase (CAT), and superoxide dismutase (SOD) in renal tissue.

***Transmission electron microscopy***

The renal tissue was fixed for 2 hr before being placed in 2% glutaraldehyde, and fixed for 2 hr after 1% starved acid. The gradient ethanol was dehydrated in turn, epoxy propane was infiltrated, the tissue was embedded, and the sections were made. The ultrastructure of the kidney tissue was observed under a transmission electron microscope (HIT-ACHI H7650, Nishizaki Technology Co. Ltd, Japan).

***Western-blotting***

Renal tissue was weighed and protein lysate (Wuhan Sanying Biotechnology Co., Ltd., Wuhan, China) was added to extract the protein. Samples were loaded in proportion. Protein was separated by polyacrylamide gel electrophoresis, wet transferred to PVDF membrane at low temperature, soaked in the blocking solution containing 5% skimmed milk for one hour, and then NLRP3 antibody (1:500), caspase-1 antibody (1:1 000) (Guangzhou Co, Guangdong, China), apoptosis-associated speck-like protein containing a caspase recruitment domain foci (ASC) were added β Antibodies (1:2000) (Guangzhou Co., Guangdong, China), and GAPDH antibodies (1:2000) (Guangzhou Co, Guangdong, China) were placed at 4 ℃ overnight, washed with Tris buffer solution three times the next day, incubated in a horizontal shaker with anti-II for one hour, and exposed for sampling after film washing. ImageJ software (Version 1.8.0, Bharti Airtel Ltd, India) was used for analysis. 

***Immunofluorescence***

The kidney tissue was fixed with 4% paraformaldehyde for 24 hr, and then the sections (2–3 µm) were incubated with a sealed buffer solution for 30 min. The diluted NLRP3 antibody (1:1000) (Invitrogen Co, Shanghai, China) was added, and incubated at room temperature for one hour, then washed with buffer solution 3 times. The second antibody (Invitrogen Co, Shanghai, China) was added, and incubated at room temperature for 30 min, then fixed with a prolonged anti-fading agent with 4 ‘, 6-dimid-2-phenylindole (Invitrogen Co, Shanghai, China) and observed under a fluorescence microscope.

***RT-qPCR***

Total RNA was extracted from renal tissues using RNA simple total RNA extraction kit (Boster Co., Wuhan, China). RT-qPCR method was used to determine the mRNA expression level in renal tissue. Reaction parameters: the PCR condition was 95 °C for 10 min, and then 40 extended cycles: 95 °C for 20 sec, and 60 °C for 75 sec. β-actin was selected as the internal reference, and 2 ^− ΔΔCt ^was used to calculate the relative level of target mRNA.

***Statistical analysis***

Prism Graphpad 6.02 (San Diego, California, USA) was used for statistical analysis. The data were expressed by mean±SD or median (IQR). We used one-way ANOVA to compare data with normal distribution and homogeneous variance and compared the two groups by Tukey *post hoc* test. *P*<0.05 was statistically significant. 

## Results

***Protective effect of SNG on AKI in septic rats***

**Figure 1:**
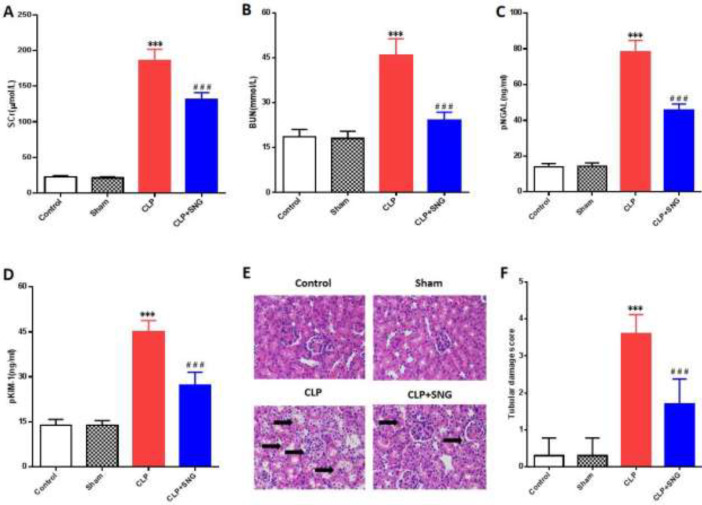
Protective effect of SNG on AKI in septic rats. (A) level of SCr. (B) level of BUN. (C) level of pNGAL. (D) level of pKIM-1. (E) pathological damage of kidney tissue (H&E, 400×). (F) pathological score of kidney tubular epithelial tissue

To determine the protective effect of SNG on AKI in septic rats, we first measured the levels of renal function indicators. We found that CLP induced an increase in pKIM-1, pNGAL, BUN, and SCr levels in septic rats (all *P*<0.001), accompanied by a decrease in urine volume (Figure 1A-D). However, after pretreatment with SNG, the levels of pKIM-1, pNGAL, BUN, and SCr in septic rats were significantly reduced (all *P*<0.001) (Figure 1A-D). Then, we used H&E staining to evaluate the renal histopathological changes. As shown in Figure 1E, the renal tissue structure of rats in the Control and Sham groups was complete and clear, and no obvious changes in renal tubule damage were found. The vacuolar degeneration of renal tubule epithelium in rats induced by CLP was aggravated, and interstitial hemorrhage and inflammatory cell infiltration were scattered. However, pretreatment with SNG could reduce the damage of renal tubular epithelial cells (RTECs) interstitial hemorrhage and inflammatory cell infiltration. Through semi-quantitative analysis, we found that SNG significantly reduced the CLP-induced renal tubular epithelial injury score (*P*<0.001) (Figure 1F). The above results confirmed that SNG had a protective effect on AKI in septic rats. 

***Anti-inflammatory and anti-oxidative effects of SNG on septic AKI***

**Figure 2:**
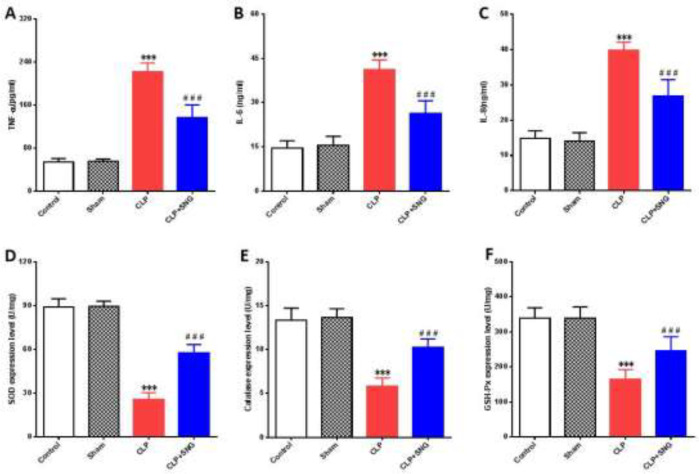
Anti-inflammatory and anti-oxidative effects of SNG on AKI in septic rats. (A) level of TNF-α. (B) level of IL-6. (C) level of IL-8. (D) level of SOD. (E) level of CAT. (F) the level of GSH-Px

SNG: s-nitroso glutathione; AKI: acute kidney injury; SOD: superoxide dismutase; CAT: catalase; GSH-Px: glutathione peroxidase

Previous studies indicated that SNG significantly improves the prognosis of septic rats, and has anti-inflammatory and anti-oxidant effects (8, 10). To determine the anti-inflammatory and anti-oxidant effects of SNG on AKI of septic rats, we detected the levels of TNF-a, IL-6, and IL-8 in renal tissues, and also measured the levels of GSH-Px, CAT, and SOD. We found that the levels of TNF-a, IL-6, and IL-8 were increased in the renal tissues of septic rats (all *P*<0.001) (Figure 2A-C), accompanied by a decrease in the levels of SOD, CAT, and GSH-Px (all *P*<0.001) (Figure 2D-F). However, SNG pretreatment could significantly reverse the above phenomena (all *P*<0.001), which indicated that SNG had significant anti-inflammatory and anti-oxidant effects on the renal tissue of septic rats. 

***Protective effect of SNG on mitochondria of RTECs in septic rats***

**Figure 3:**
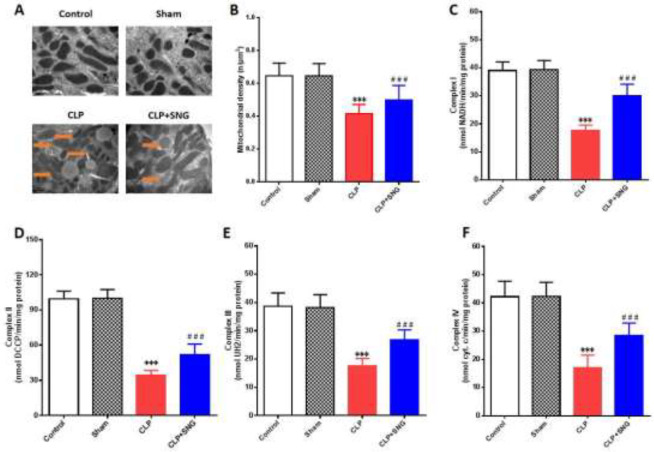
Protective effect of SNG on mitochondria of RTECs in septic rats. (A) mitochondrial structure changes of RTECs. (B) quantitative determination of the average mitochondrial density (n/μm3) in RTECs. (C) level of Complex I. (D) level of Complex II. (E) level of Complex III. (F) level of Complex IV

SNG: s-nitroso glutathione; RTECs: Renal tubular epithelial cells

The damage of intracellular mitochondria and dysfunction of energy metabolism caused by severe infection are the focus of current research, but the specific molecular mechanism is still unclear (11-13). To clarify the protective mechanism of SNG on mitochondria of RTECs, we measured the morphology and density of mitochondria of RTECs and the level of mitochondrial enzyme complex I/II/III/IV in rats. We found that CLP induced the dissolution of RTECs mitochondria in septic rats (*P*<0.001) (Figure 3A), while the mitochondrial density (Figure 3B) and the level of enzyme complex I/II/III/IV decreased significantly (all *P*<0.001) (Figure 3C-F). However, SNG improved the mitochondrial morphology of RTECs, accompanied by a significant increase in mitochondrial density and enzyme complex I/II/III/IV level (all *P*<0.001) (Figure 3A-F). Our results confirmed that SNG could significantly protect AKI from sepsis by improving the morphology and function of RTECs mitochondria.

***SNG inhibits the pyroptosis of RTECs in septic rats***

**Figure 4.**
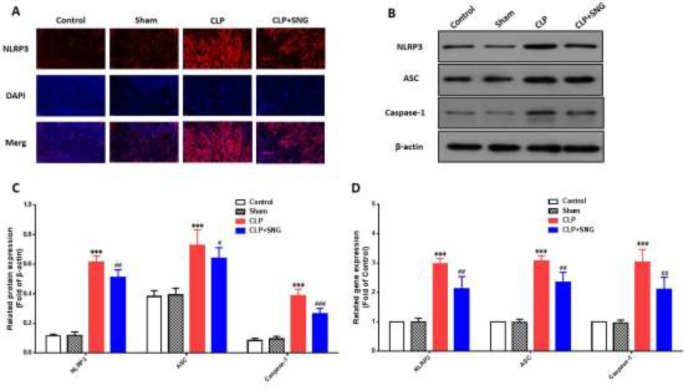
SNG inhibits the pyroptosis of RTECs in septic rats. (A) expression of NLRP3 in renal tissue. (B) expression of pyroptosis pathway proteins. (C) relative protein expression levels of the pyroptosis pathway. (D) relative mRNA expression of the pyroptosis pathway

SNG: s-nitroso glutathione; RTECs: Renal tubular epithelial cells; NLRP3: the NOD-like receptor protein 3

Pyroptosis is a hot topic in sepsis research. To investigate whether the protective mechanism of SNG on septic AKI is related to the pyroptosis of RTECs, we measured the protein expression of the pyroptosis pathway. Firstly, we used immunofluorescence to observe the expression of NLRP3 protein in the renal tissues of rats. We observed that the expression of NLRP3 was increased in the renal tissues of septic rats, and SNG pretreatment could reverse this phenomenon (Figure 4A). Secondly, we measured the levels of pyroptosis pathway proteins by western blotting, and we found that the levels of NLRP3, ASC, and caspase-1 were all significantly increased in septic AKI (all *P*<0.001), while SNG reduced the expression of these proteins (all *P*<0.001) (Figure 4B-F). Finally, we used RT-qPCR to measure the mRNA of NLRP3, ASC, and caspase-1. Similar to the above experimental results, the mRNA of NLRP3, ASC, and caspase-1 were all increased in septic AKI (all *P*<0.001), and SNG pretreatment reversed the above phenomenon (all *P*<0.05) (Figure 4G). Our above experiments confirmed that SNG had a protective effect on septic AKI by inhibiting the pyroptosis pathway of RTECs. 

**Table 1:** The mRNA sequence used in this study is as follows

**Name**	**Direction**	**Sequence**
NLRP3	Upstream	5 '- ACT CCC GTG CAT CGC CTA GCC AG-3'
	Downstream	5 '- GCC GTC CAT CAG CCA GCT GAT AGC TC-3'
ASC	Upstream	5 '- CTG ACC ATT CCA TGT CAT CCA CAT-3'
	Downstream	5 '- ACG ACC ATC CTG TGA AAG CTA GC-3'
Caspase-1	Upstream	5 '- CAA GCC CGT GTA CAA CGC CTG ATC CG-3'
	Downstream	5 '- GGC ACA CCG ACA CGT CAG CGT CAC-3'
β-actin	Upstream	5 '- CAG AAC GCC GCC CGT GTC AC-3'
	Downstream	5 '- CAC GCT GCA TCC CGA ATC GC-3'

SNG: s-nitroso glutathione; AKI: acute kidney injury; SCr: serum creatinine; BUN: blood urea nitrogen; pNGAL: plasma neutrophil gelatinase-associated lipocalin; pKIM-1: plasma kidney injury molecule-1

The data were expressed as mean±SD. Compared with the Sham group, ***P<0.001; Compared with the CLP group, ###*P*<0.001

The data were expressed by mean±SD. Compared with the Sham group, ***P<0.001; Compared with the CLP group, ###*P*<0.001

The data were expressed by mean±SD. Compared with the Sham group, ***P<0.001; Compared with the CLP group, ###*P*<0.001

The data were expressed as mean±SD. Compared with the Sham group, ***P<0.001; Compared with the CLP group, #*P*<0.05, ##*P*<0.01, and ###*P*<0.001

## Discussion

AKI is a common complication of sepsis, which is independently related to the increase in mortality of patients (14, 15). In animal experiments, CLP-induced sepsis is the most widely used method for preparing models of sepsis (16). In our study, the pathological changes induced by CLP in septic rats mainly focused on renal tubule damage, including tubular epithelial cell vacuolar degeneration, renal tubule expansion, mitochondrial damage, and even epithelial cell apoptosis. After SNG intervention, the degree of renal tubule damage was reduced, indicating that SNG can improve the renal pathological damage of AKI in rats with sepsis to a certain extent. 

As the traditional indicators for the diagnosis of AKI, SCr, and BUN mainly reflect the filtration function of the glomerulus, while pNGAL and pKIM-1, as effective markers for the diagnosis of early AKI, mainly increase significantly when the renal tubules are injured (17). In the present study, SNG pretreatment reduced the levels of pKIM-1, pNGAL, BUN, and SCr, which suggested that SNG could improve the glomerular filtration function of septic rats and reduce the damage of renal tubules.

The pathogenesis of AKI caused by sepsis is complex and diverse, among which ischemia-reperfusion injury is an important factor leading to AKI, which is mainly regulated by oxygen free radicals, various cytokines, and chemical molecules (18). Chen *et al*. (19) studied and analyzed cells, and found that during renal ischemia/reperfusion, the intracellular reactive oxygen species increased, but the endogenous anti-oxidants did not increase accordingly, which led to the accumulation of reactive oxygen species in the cells, thus damaging the cells. In the process of inflammatory reaction, the oxygen free radicals produced in the body will increase explosively, which is difficult to be cleared by SOD, CAT, GSH, and other anti-oxidant enzymes in the body, thus leading to the accumulation of active oxygen clusters in the kidney tissue, the level of inflammatory factors will increase, and the degree of organ damage will increase (20).

SNG, a small S-nitroso glutathione and a synthetic substance of NO reduces the level of iNOS and improves renal microcirculation (8). In recent years, studies showed that SNG can inhibit the production of inflammatory factors, reduce the inflammatory response, inhibit the activation of the apoptotic signal pathway, and protect the kidney, lung, and other important organs (7, 8). Moreover, SNG reduces IL-6, IL-1β, and TNF-α levels in the lung tissue of septic mice, which has a protective effect on septic acute lung injury (21). In our study, we found that SNG pretreatment could improve renal function, reduce the inflammatory response and oxidative stress, and significantly protect against AKI in sepsis. 

Lysosome is an important organelle in cells, which is closely related to mitochondria (22). Oxidative stress induces peroxidation of the lysosomal membrane, increases lysosomal membrane permeability, and releases hydrolases and apoptotic factors (23). Meanwhile, the damaged mitochondria produce a large number of oxygen free radicals, which aggravates the damage to lysosomes, and forms a chain cycle with lysosomes and mitochondria as the axis (24). The results of this study showed that the mitochondria of RTECs in septic rats were damaged, which showed swollen mitochondria, unclear membrane structure, transparent matrix, and broken or even dissolved cristae. Moreover, mitochondrial function was impaired, which showed that the density of mitochondria and complex enzymes in RTECs was decreased. After the pretreatment of SNG, the ultrastructure and function of mitochondria in RTECs were significantly restored, and the density of mitochondria and the level of complex enzymes were significantly increased, indicating that SNG could improve the mitochondrial function of RTECs in septic rats.

In recent years, studies showed that NLRP3 inflammatory corpuscles are the key link of inflammatory response and have important significance in many inflammatory diseases (24, 25). The activation of NLRP3 inflammatory bodies has two stages. One is to activate the nuclear transcription factor signaling pathway and mediate the production of IL-1β by inducing Toll-like receptor-4 and IL-18 precursor; Second, lipopolysaccharide and other stimulus signals combine with NLRP3 receptor to promote the secretion of mature IL-1β and IL-18, thereby exerting immune inflammatory effect (26). Studies have shown that RTECs also express TLR4 receptors, and inflammatory mediators directly or indirectly damage RTECs, causing mitochondrial damage, energy metabolism disorder, and even apoptosis (27). 

In our study, the expression of NLRP3, ASC, and caspase-1 in renal tissue of the CLP group increased significantly, and showed a progressive increase trend with the prolongation of postoperative time, which was consistent with the degree of renal pathological damage, indicating that the stronger the renal inflammatory reaction, the more severe the renal pathological damage, and the worse the renal function. SNG pretreatment significantly reduced the levels of NLRP3, ASC, and caspase-1, and suggested that SNG could reduce the release of inflammatory factors by inhibiting the activation of NLRP3 inflammatory bodies, thereby reducing the inflammatory reaction of renal tissue and improving the pathological damage of renal tubules and renal function.

## Conclusion

SNG pretreatment improved the renal function and pathological changes of CLP-induced septic rats, reduced the level of inflammatory reaction and oxidative stress in renal tissue, and improved the morphology and function of mitochondria. The specific mechanism is closely related to the inhibition of the activation of NLRP3 inflammasome. 

## Authors’ Contributions

HF, MS, and JWL performed the experiments, interpreted of data, and wrote the first draft of the manuscript. JHZ and HF participated in the conception, design, and providing critical revisions. All authors read and approved the final manuscript.

## Conflicts of Interest

No conflicts of interest to declare. 
